# Exploring healthcare staff narratives to gain an in-depth understanding of changing multidisciplinary team power dynamics during the COVID-19 pandemic

**DOI:** 10.1186/s12913-023-09406-7

**Published:** 2023-05-01

**Authors:** Lisa Rogers, Aoife De Brún, Eilish McAuliffe

**Affiliations:** University College Dublin Centre for Interdisciplinary Research, Education, and Innovation in Health Systems (UCD IRIS), School of Nursing, Midwifery and Health Systems, Dublin, Ireland

**Keywords:** Power, Hierarchy, Healthcare, Multidisciplinary care teams, COVID-19

## Abstract

**Background:**

Multidisciplinary teams (MDTs) are integral to healthcare provision. However, healthcare has historically adopted a hierarchical power structure meaning some voices within the MDT have more influence than others. While power dynamics can influence interprofessional communication and care coordination, the field’s understanding of these power structures during the COVID-19 pandemic is limited.

**Methods:**

Adopting a narrative inquiry methodology, this research addresses this knowledge gap and provides an in-depth understanding of MDT power dynamics during COVID-19. Using semi-structured interviews (n = 35) and inductive thematic analysis, this research explores staff perspectives of changing power dynamics in MDTs during the pandemic response.

**Results:**

An in-depth analysis generated three overarching themes: (1) *Healthcare: a deeply embedded hierarchy* reveals that while a hierarchical culture prevails within the Irish health system, staff perceptions of influence in MDTs and ‘real’ experiences of autonomy differ significantly. (2) *Team characteristics: the influence of team structure on MDT power dynamics* highlights the impact of organisational structures (e.g., staff rotations) and local processes (e.g., MDT meeting structure) on collaborative practice. (3) *Ongoing effort to stimulate true collaboration* underscores the importance of ongoing interprofessional education to support collaborative care.

**Conclusion:**

By offering a greater understanding of MDT power dynamics throughout the COVID-19 pandemic, this research supports the development of more appropriate strategies to promote the provision of interprofessional care in practice.

**Supplementary Information:**

The online version contains supplementary material available at 10.1186/s12913-023-09406-7.

## Background

Within healthcare, multidisciplinary teams (MDTs) are an integral feature of care provision. By valuing the skills and knowledge of each discipline, holistic patient-centred care can be achieved [1, 2]. However, interprofessional collaboration is challenging. Despite the promotion of interprofessional care in research and policy, implementation of this egalitarian model is often absent in practice [3]. Each professional group has a unique identity that corresponds to their discipline-specific training and clinical experience [4, 5]. Within the Irish health system (the context of this research) while the latest reform focuses on the need for developing the “right team” to support interdisciplinary team-based working [6], currently HCPs are trained intraprofessionally in discipline- specific groups. This uni-professional approach socialises HCPs to function independently and autonomously within their profession [7]. The distinct identity of each discipline means that despite sharing the same goal of improving patient outcomes, healthcare professionals (HCPs) may have differing priorities and expectations about how care should be delivered [8–10]. These diverse interests can result in HCPs working in discipline-specific silos (nursing, medicine, allied health), where professions leverage their discipline specific knowledge to strengthen their value within the MDT [8]. For example, if only one particular profession can define and solve a problem, this unique knowledge prevents outside interference or control. Therefore, knowledge is critical in establishing power in MDTs. However, traditional norms of organisations mean that some HCPs’ voices have more influence than others [11].

Healthcare has historically been characterised by a hierarchical power structure with physicians assuming dominant roles [12, 13], while other professions encounter challenges establishing their status in terms of patient care decisions [8]. During COVID-19, some researchers report softer hierarchies within MDTs [14, 15], while others note that care decisions throughout the pandemic response have reportedly shifted back towards a hierarchical model [16]. Engum and Jeffries [17] suggest that imbalances of power between professions can influence communication, the coordination of care and ultimately patient safety. Stereotypes such as doctors being primary leaders, while other professions remain passive in patient care decision-making, can create divisions within the team, limiting interprofessional collaboration [10]. The absence of team-based care can adversely affect the quality of service provision as patients may potentially fall through the system’s cracks [18]. Despite the reported impact of power dynamics, hierarchical structures in healthcare are rarely explicitly discussed [19]. The absence of this discourse suggests a hesitancy to acknowledge the realities of hierarchy in healthcare or a failure to question the status quo. To increase collaborative practice, power disparities among MDT members must be understood and made explicit to establish targets for change. This research contributes new insights by providing an in-depth understanding of changing MDT power dynamics during the COVID-19 pandemic.

## Methods

### Study Design

To understand healthcare professionals’ real-life experiences of power dynamics within healthcare teams during the COVID-19 pandemic, this research adopted a narrative inquiry methodology. This approach uses story-telling to communicate participant realities and provides a rich, in-depth description of experiences [20].

### Study sample

Multidisciplinary team members from a diverse range of professions (i.e., doctors, nurses, allied health professionals (AHPs), support staff) and team types (i.e., specialty and location) were invited to participate via local and national gatekeepers within the Irish health system. Following the identification of participants, snowball sampling through word-of-mouth was used. Due to ongoing COVID-19 surges and pressure on healthcare staff during data collection, nurses were underrepresented (n = 3) and support staff (e.g., healthcare assistants) were absent from the study sample. However, overall sample adequacy was achieved during the interview process as a sufficient depth of information was gathered to produce no new information or new thematic patterns from the data collected.

**Table 1:** Characteristics of study participants

Participant	Sex	Years’ experience	Sample details
Nurse01	F	20 years	Sample included nurses and nurse managers from mental health (n = 1), public health (n = 1), and acute care (n = 1) settings.
Nurse02	F	22 years	
Nurse03	F	8 years	
Midwife01	F	12 years	Sample comprised of midwifery managers
Midwife02	F	35 years	
Med01	M	5 years	Sample encompassed non-consultant hospital doctors (senior house officers (n = 3) and registrars (n = 5)), consultants (n = 3) and an orthodontist (n = 1).
Med02	F	6 years	
Med03	F	6 years	
Med04	F	7 years	
Med05	M	1 year	
Med06	M	1 year	
Med07	F	1 year	
Med08	F	3 years	
Med09	F	16 years	
Med10	M	≈ 30 years	
Med11	M	30 years	
Med12	F	> 20 years	
AHP01	F	3 years	Sample contained various disciplines from the field of allied health (i.e., physiotherapy (n = 4), occupational therapy (n = 1), social work (n = 7), speech and language therapy (n = 1), pharmacy (n = 1), radiography (n = 1), psychology (n = 3))
AHP02	F	22 years	
AHP03	F	3 years	
AHP04	F	20 + years	
AHP05	F	23 years	
AHP06	F	1 year	
AHP07	M	35 years	
AHP08	M	9 years	
AHP09	F	10 years	
AHP10	F	5 years	
AHP11	F	1 year	
AHP12	F	16 years	
AHP13	F	> 15 years	
AHP14	F	9 years	
AHP15	F	3 years	
AHP16	F	19 years	
AHP17	F	17 years	
AHP18	F	7 years	

### Data collection

To elicit staff narratives and gain an in-depth understanding of healthcare staff experiences of MDT power dynamics, semi-structured interviews were conducted between September 2021 and March 2022. The interview schedule was piloted once, resulting in minimal changes to the structure. This pilot interview was included in the final dataset. 35 participants from across the Irish health system (i.e. three nurses, two midwives, twelve doctors, and eighteen AHPs) were interviewed once (Table 1), and interviews ranged in duration from 32 to 68 minutes. The researcher (LR) conducting the interviews had no prior relationship with participants. Interviews were conducted remotely using telephone calls and the videoconferencing service Zoom. All interviews were audio-recorded and transcribed verbatim (see Supplementary material 1 for topic guide).

### Data analysis

Thematic analysis as outlined by Braun and Clarke [21] guided the analysis. This process involved repeatedly reading the data, generating initial codes, and developing, refining, and naming broader themes. Rather than applying a prescriptive list of codes, an inductive approach to coding was applied which ensured themes strongly reflected the data collected. Using NVivo12 software, an experienced qualitative researcher (primary researcher LR) conducted line-by-line thematic coding of each interview transcript. The relationship between these initial codes were explored and where appropriate codes were combined to develop broader themes. As the primary researcher’s previous experience as a registered nurse likely impacted their interpretation of the data, these themes were deliberated and refined through discussions with the research team. The dependability of the findings was further enhanced through deviant case analysis [22]. Recognizing alternative viewpoints achieved a more holistic understanding of the data.

### Ethics

An ethical exemption was granted from the University College Dublin Research Ethics Committee (ref: HREC-LS-E-21-186) due to the low-risk nature of this research. All participants provided written informed consent and all potentially identifiable characteristics were removed from each transcript to maintain anonymity.

## Results

Three key findings were generated from the inductive analysis: (1) Healthcare: a deeply embedded hierarchy (2) Team characteristics: the influence of team structure on MDT power dynamics, and (3) Ongoing effort to stimulate true collaboration. Data are presented using participants’ professional group (e.g., AHP01). Table 1 presents additional information to assist the reader in contextualising the findings. Table 2 provides a summary of each theme with exemplar quotes and study recommendations.

### Healthcare: a deeply embedded hierarchy

Most staff confirmed that a hierarchical power structure remains deeply engrained within the Irish health system with senior physicians described as the primary decision-makers within most MDTs. However, staff perceptions of who holds power versus the reality of who exercises influence varied across team members. COVID-19 impacted team power structures by stimulating more collaborative or siloed working amongst diverse team types (i.e., acute vs. community services). The sub-themes of (1) *Physician authority*; (2) *Factors influencing hierarchical decision-making;* (3) *Perceptions of influence vs. experienced power*; and (4) *COVID-19 exposing contrasting experiences of interprofessional care* explain these findings.

#### Physician authority

Most participants described the decision-making processes within their respective MDTs as hierarchical. Participants portrayed the Irish health service as an “old fashioned” system (AHP15) where medical dominance dictates care decisions. Staff from both acute and community services considered the influence of their medical colleagues to be “almost like a God” (AHP01). Many participants emphasised that care delivery centres around a medical model meaning that physicians remain “first in command” (AHP11), while others have a “deference” (Med10) towards this authority. AHPs identified a divide between medical and therapeutic professions. This division was predominantly associated with the diverse priorities underlying decision-making processes (i.e. medical vs. social model). Some AHPs voiced the need to develop “alliances” (AHP08) with other disciplines, specifically nursing and other AHPs. Gaining this “backup” (AHP15) was important to strengthen their voice within the MDT and enhance their influence on care decisions. Participants from acute care settings highlighted that the structure of care delivery where patients are admitted under the care of one senior physician further encourages hierarchical decision-making: “we do not discharge people, we do not take people- it is under a different person’s name” (AHP15). Participants from all professions emphasised that due to this structure, senior physicians have the “final say” (Med01) and hold ultimate responsibility. All disciplines considered hierarchical decision-making as a necessity: “the buck has to stop at someone, if there’s no hierarchy, then no one’s responsible” (Med04). For some, hierarchical decision making provided a level of comfort:

“it’s nice to kind of have this… like a parental figure, that kind of ‘go to’ person where at the end of the day the buck does stop with them” (AHP01).

Acceptance for the status quo was reflected in some staff experiences of collaborative leadership styles: “the consultant was very equal and actually then he was criticized that he could never make a decision” (Nurse01). Senior physicians also identified challenges in changing culture and the hierarchical traditions within their MDTs: “I ask everyone to call me my first name and they never do” (Med11).

#### Factors influencing hierarchical decision-making

Physician attitudes were cited as influential in shaping and reinforcing the culture of a MDT. While some physicians created boundaries by using professional titles, others “encouraged feedback” (Med04), and prioritised strengthening relationships within their MDTs:

“the doctors did a lunch for the nurses…there’s that respect because we all do work very closely together all the time” (Med12).

Many cited physician speciality as a critical factor influencing attitudes towards decision-making processes. Surgeons were characterised as hierarchical leaders. Participants associated this approach with the acute and less “ambiguous” (Med08) nature of the surgical specialty:

“they’re like carpenters- they come in, they have something to fix, they fix that thing, and then move on to the next person…” (AHP10).

However, due to disease and patient complexity, some surgical subspecialties (e.g. breast and vascular) warranted a more collaborative approach:

“vascular there’s huge MDT input because patients have such drastic changes to their lives… {after} an amputation it’s really physio, OT, and nursing staff who are much more important after the operation in terms of getting a patient to a new baseline” (Med07).

Other complex specialities characterised as collaborative in nature included medicine for the older persons, paediatrics, emergency medicine, and critical care. Physicians within these services were acknowledged as understanding the value of working collaboratively to ensure the provision of optimum person-centred care. Within mental health services greater collaboration assisted with risk management. By sharing responsibility across the MDT, high risk cases could be better controlled or “contained” (AHP14). However, staff also suggested that some cases need “a firm hand” (Nurse01) to ensure staff and patients are clear in relation to who has the final say:

“…top down actually worked because we all needed somebody to say this is the way it is… we might all give out for a couple of weeks and then something would happen and we’d be like thank god they actually took the lead on that” (Nurse01).

In addition to holding positional authority, other characteristics impacting staff influence within MDTs included staff knowledge, stability, and visibility. These factors promoted the value of a profession within MDTs. Due to the transient nature of some roles (i.e., input dependent on patient needs), staff strengthened their influence by “blinding people with jargon” (AHP16):

“I have learned to alter my dialect using {medical} words more than mine… to get across I know exactly what I’m saying” (Midwife01).

Personalities were also cited as an unpredictable influence. While many suggested “those that shout the loudest will be heard” (Midwife02), others distinguished that with time “the ones who listen… and have integrity” become the strongest influence as they gain the respect of their team (Med10). Reflecting these characteristics, within acute care settings, participants from all professions emphasised the “central role” (Med01) of the clinical nurse manager. In community services, staff identified the importance of strong line managers across disciplines to instil confidence in their staff: “if you’ve got good supervision you’re able to be certain with what you’re bringing to the team” (AHP14).

#### Perceptions of influence vs. experienced power

For some disciplines, perceptions of a profession’s influence differed from participant experiences of power within their respective MDTs. While all nursing, AHPs, and junior physicians perceived senior physicians as holding ultimate control, many senior physicians themselves considered senior management as the strongest influence. Senior physicians described how the “{medical} hierarchy is being exchanged for another” (Med11). While senior physicians confirmed their autonomy in terms of patient care decision-making, senior managers were cited as ultimately holding power in terms of strategic and team operations: “{new initiatives} are never really going to be successful if you don’t get that management buy in” (Med09).

For AHPs, although many felt that they had limited power within their own MDT, other professions identified the role of physiotherapists and social workers as influential to service delivery. While, this influence was predominantly described in terms of a supporting role (particularly during discharge planning), during COVID-19, staff expressed a more holistic understanding of the “vital” (Med08) role of AHPs in patient care delivery. For some, this heightened recognition resulted in greater funding, particularly for social work services:

“During COVID because of the high mortality rates and the issues around visiting and the kind of emotional impact, we were allocated some funding… {it’s} like they’ve learned through experience I suppose of what value there is to having a social worker there” (AHP02).

#### COVID-19 exposing contrasting experiences of interprofessional care

COVID-19 had a diverse impact on the decision-making processes within MDTs. Some specialities and disciplines came to the forefront in terms of their influence in the team. The pandemic was cited as “elevating the profile” (AHP07) of infection prevention and control teams. Through their leadership and education, they secured a position at “the top table” (AHP02) impacting decision-making at local and national levels. Participants also commended the role of porters, cleaners and catering staff. Although not traditionally “counted as part of the MDT” (AHP17), during COVID-19 staff recognised the value of these team members in delivering patient care:

“it wasn’t just kind of the professionals… everyone was valued, it took a lot to like run the hospital and care for the patient…” (Nurse03)

Some staff described COVID-19 as an opportunity to bond and breakdown professional barriers. Participants reported receiving greater emotional support from their MDT colleagues as “people could relate to how difficult it was” (Med06). Staff also reported undertaking additional roles to assist their colleagues. For example, one senior physician reflected on their experience “rolling patients, cleaning patients and bringing tea” (Med03). For some, changes within their organisation that affected all staff (e.g., requirement to wear scrubs) mitigated power disparities as “it felt like everyone was on the same team” (Med04). For others the uncertainty associated with the pandemic supported in flattening hierarchical structures because “no one knew better than anybody, because nobody knew anything” (AHP12). However, others described a contrasting experience where decision-making became “medicalised again” (AHP02). For staff particularly in community settings, COVID-19 restrictions and the lack of opportunities to interact reduced collaboration which resulted in people “focusing on their own work… going back into their disciplines as opposed to sharing across” (AHP14).

### Team characteristics: the influence of team structure on MDT power dynamics

Participants identified several factors contributing to the culture of their MDTs: communication processes, respect, role clarity, and psychological safety. Most cited structural factors related to the team as triggering greater collaboration (e.g. inclusive communication). The subthemes of (1) team stability and visibility, and (2) team interactions demonstrate how team structures and processes influence MDT power dynamics.

#### Team stability and visibility

Staff suggested the high turnover of team members as a central factor contributing to a lack of familiarity and role appreciation. Junior physicians highlighted that due to their frequent rotations through the health system they’ve “only just settled into a ward” (Med08) before relocating and building relationships again. For AHPs, turnover resulted in some MDT members “downplaying” the skill associated with their role:

“would you mind going up and having a chat with that person- that’s like a little red rag to a bull…there’s a few people that would think well sure I could do a bit of that” (AHP02)

By “sticking around” (AHP15) some AHPs secured the respect of their fellow team members. Staff within “static” MDTs (AHP13), confirmed that the longevity of their interactions provided opportunities to prove their worth among team members. For many, this stability established trust which created a “safe space” (AHP13) to speak up and challenge “any rank” within their team (Midwife01).

For many AHPs their “lack of presence” (AHP10) on the ward and intermittent engagement (dependent on patient need or resourcing) negatively impacted their ability to establish relationships and promote their worth within the team:

“When we just dip in and out- the value of {our role} wouldn’t be as fully known. When they have to manage without you, then they sort of quickly find ways to forget what it was you did…” (AHP07).

For staff in acute care settings COVID-19 promoted greater familiarity across professions because care delivery became ward-based. This service redesign resulted in the heightened stability, visibility and accessibility of all MDT members which subsequently enhanced role understanding and appreciation:

“team members realised how important it is to use the team and to rely on your team and you’re not just in it alone, because you couldn’t possibly get all the work done yourself” (AHP11).

Physicians in particular reported the development of more functional MDTs as “everyone on the ward knew each other” (Med07): “you’re always dealing with the same people which is good because of that familiarity and trust” (Med08). The uncertainty and shared experience of working during the pandemic further strengthened MDT relationships for many: “like an army unit that goes into battle, you come out and you’re probably closer” (Med11). However, remote working was mandated for many community AHPs which resulted in the “split” of MDT members:

“unless you’re physically up there and you’re physically in the same building and you’re physically at the meetings with {physicians and nurses}, you sort of get maybe a little bit forgotten about” (AHP16).

Some staff also described an “unsaid” tension (AHP14) that existed between professions since COVID-19. Differing expectations in terms of the risk associated with different roles was related to a profession’s visible contribution to care on the frontline. These staff perceptions were cited as straining some MDT relationships which may impact power relations between professions moving forward:

“I suppose in the height of it all the nurses were getting frazzled like we’re the ones stuck in PPE day in, day out and then {AHPs} would ring with something that seemed unreasonable…so there was a bit of tension there definitely… you perceived it as well {AHPs} are just swanning around, we’re actually dealing with positive cases and dealing with the frontline” (Nurse01).

#### Team interactions

Most participants identified frequent MDT meetings and/or huddles as essential for enhancing collaborative team working, and ultimately patient care delivery. While these meetings provided a dedicated time to gain consensus across the team about care planning, the structure of these meetings/huddles impacted their effectiveness. Some participants described needing a senior physician “to drive” the meeting (AHP05) due to their influence within the team. Others suggested the location and arrangement of the room as influential to ensure “everyone’s had their input… and gets a turn at the table” (Nurse01):

“we all sit in a big circle, and everyone comes in, they say hello to each other and you feel a lot more comfortable… in other {meetings} they’re absolutely rushing. They just want to fly through them and it’s a very structured, kind of almost hierarchal the way that the cases are discussed” (Med03).

Many confirmed that when an MDT meeting centres around efficiency, collaboration is overlooked:

“ I think people have come to the meetings with a decision and it’d be rare enough for it to be like well, can we think what this person needs together as a group… there isn’t space to allow uncertainty. It can be really difficult going into a meeting and say I don’t know what to do with this person, it’s quite vulnerable doing that, but no I wouldn’t say it’s collaborative…it’s functional. (AHP14)

Pandemic restrictions negatively impacted team interactions. During the initial COVID-19 response, MDT meetings and huddles stopped. Some physicians were cited as embracing this change. By limiting MDT input, some perceived this change as improving the efficiency of decision-making processes. However, reduced MDT interactions was described as a “huge loss” (AHP02) for many staff in terms of their ability to promote their voice within the MDT. While MDT meetings returned to online platforms as COVID-19 progressed, the focus of these meetings shifted towards more functional, “appointment based” communication (AHP10): “it’s like what’s the plan, what’s the action, as opposed to maybe a space to think” (AHP01). Others identified a “refusal” (AHP08) or a lack of motivation from some senior team members to engage in MDT meetings throughout the pandemic:

“it was just like a nightmare, we were literally well over 14 months into it and we were still struggling to get everyone onto the meeting” (AHP16).

Staff also advised that “a lot of teamwork occurs in the corridor” (Midwife02) outside of these formal meetings. While MDT meetings support in developing “formal…polite, civil” relationships (AHP01), informal interactions promote more open dialogue and “keep {MDT} relationships in a healthy position” (AHP05) as staff get to know each other on a more personal level. COVID-19 restrictions stopped these informal check-ins and nights out which impacted interdisciplinary communication and morale:

“you don’t have the opportunity to go and sit and have a cup of coffee and often those are the things that actually make professional communication easier” (AHP02).

### Ongoing effort to stimulate true collaboration

When reflecting on the future of MDT working most staff aspired for greater inclusivity and mutual respect within their MDTs. “Having a voice that’s heard” (AHP08) and holding “equal stature” (Midwife01) were cited as central to improve staff wellbeing and mitigate the psychological consequences of the pandemic response (i.e., fatigue, burnout, stress leave):

“the team provides a resilience and it provides a comfort and it provides a camaraderie that makes work easier…more meaningful and joyous” (Med10).

Participants identified interdisciplinary education and training as important strategies necessary to promote and sustain interprofessional collaboration in MDTs. Staff recommended introducing interdisciplinary modules to the undergraduate curriculum to enhance role understanding and appreciation from the outset of a HCP’s career:

“if you’re going to be working in a MDT you need to know what do they do? why are they there? what is their value to the team” (AHP03)

Some participants hypothesised that interdisciplinary education may enhance the accessibility of staff and instil a more collaborative culture on the frontline:

“I think that starts in colleges and universities, because if they felt like please approach us, please talk to us, you know if you’ve got any questions ask me it might just be a little bit smoother…{not} just sticking a {referral} form in a box and not knowing if it’s appropriate or not” (AHP10)

However, interprofessional collaboration was described as requiring a “constant effort” (AHP13) from all team members. Therefore, staff advised that formal interdisciplinary training needs to continue throughout their career. Some recognised that teams primarily focus on the patient or client and “don’t give time to themselves… to stand back and look at all they’ve achieved… and areas that they can improve” (AHP09). Team development days were a strategy considered to offer a “safe space” (Nurse01) to learn “softer skills” (Midwife01) as a team. However, some questioned senior management buy-in and the resources needed to deliver this development training:

“what are they doing having fun…some people might think that it’s a waste of time” (AHP18).

“I really can’t see that happening, unless we hire twice the doctors and three times the amount of midwives we have at the moment to learn these softer skills” (Midwife01).

**Table 2:** Summary of developed themes and recommendations

Theme	Quotation	Recommendations
**Healthcare: a deeply embedded hierarchy** *Subthemes* 1. Physician authority 2. Factors influencing hierarchical decision-making 3. Perceptions of influence vs. experienced power 4. COVID-19 exposing contrasting experiences of interprofessional care	1. “…we are still very much in a medical model, so the attitude and behaviour of the consultant is probably a really big factor in terms of how multidisciplinary teams work” (AHP02) “doctors will tend to kind of, I suppose, they are first in command and it’s what they say kind of goes… it’s a historical issue. I think it’s probably just a structural thing within the teams here, ultimately, people are in an acute setting because of medical issues” (AHP11)	The governance of care must be reviewed and policies introduced to instil interprofessional decision-making irrespective of team context (e.g. team location (i.e. acute vs. community) or speciality). As healthcare contexts are dynamic and continuously changing, future research should endeavour to explore the evolution of perceptions of power within MDTs.
	2. “So I think pressure, probably makes diamonds and that’s perhaps where you know team being the diamond in the emergency department we’re very reliant on it and we’re very reliant on team members” (Med10) “…it often comes down to the specialty of the consultant. So we would find that you know maybe care of the older person would have a much greater understanding of the role that others play in overall patient care. I think the surgical disciplines tend to be much kind of cleaner- we go in, we take out whatever we need to take out, we operate, we do whatever and after that they’re less involved, I guess and it’s sort of like, look, you know what’s going on for the patient outside of here doesn’t really have any bearing in terms of their decision making” (AHP02)	
	3. “{senior physicians} are the most influential…there’s no denying that there’s a huge positional difference… there’s a vast space between a consultant, and everybody else sitting at the table” (AHP06) “I suppose there is that sort of hierarchy and even though you learn about organizational change and culture and you’re kind of tiptoeing around it and trying to kind of make it a win, and kind not step on egos and ask for advice and look for engagement {from senior managers} ehm because it’s never really going to be successful if you don’t get that management buy in” (Med09)	
	4. *Community* “for the first few months, ehm there was there was absolutely no communication, so I didn’t have access to you know, like say group telephone, you know the conference calls. So it was very much on an individual basis, and it kind of nearly felt for a while that you were holding the risk because you hadn’t a clue what was going on with the other team members, you hadn’t a clue… you know even in terms of the clients treatment plan it’s like everything just stopped” (AHP01) *Acute* “our team definitely bonded and got closer…especially in the early days of COVID like in March, April we were looking I guess at the TV and looking at scenes of Europe and other countries and thinking is this going to happen to us, so we very much just supported each other, you know because it was emotional some days and you really needed support and I felt like we all supported- it doesn’t matter who it, whether it was the cleaners, or the porters we all helped each other and supported each other so yeah I think definitely COVID did strengthen that relationship with everyone in the hospital” (Nurse03)	
**Team characteristics: the influence of team structure on MDT power dynamics** *Subthemes* 1. Team stability and visibility 2. Team interactions	1. “…teams went kind of ward based for a lot of COVID, which was nice because you got to know people on a ward, and you were stuck with the same nursing staff, the same physiotherapist, the same occupational therapist, same social worker, same SLT, same dietetics, same chaplain for one ward which I think a lot of people did enjoy as opposed to traipsing around different sides of the hospital like it could be 20 min from one ward to another and you don’t know anyone up there and it’s just, it’s kind of more difficult. So I don’t know, ward based care probably did ehm have a positive impact” (Med05) “we can be little bit isolated in that we get a list of patients to X Ray through the radiology information system and we could be just working away on those patients all day, all long without a lot of interaction or a lot of interaction with other disciplines unless they’re kind of coming to us directly for something. So it’s easy for us, I feel, to kind of be quite cut off unless you go out there and make those interactions happen” (AHP05)	Future research should endeavour to assess the feasibility of implementing ward-based care models to promote greater interprofessional collaboration.
	2. “the textures and the qualitative components of how we work within our professional domains, I think, is lost by the fact that we don’t meet. Like when you hear a colleague discuss even discuss a case whether it’s in terms of them describing you know their understanding of the case or the formulation of the work they’re doing you just learn a lot intuitively from that and there’s a lot of the qualitative understanding of one another’s roles. I think that’s kind of absorbed in those team meetings and in the absence of that, there’s much more sense of well again it’s like private practitioners in a sense” (AHP08). “a massive, massive kind of effect or impact of COVID on that has been that we haven’t been able to meet as a team and kind of bond our relationships…” (AHP11) “MDTs are still via zoom which is quite problematic… there isn’t as much of a flow in the interaction” (AHP17)	Development of local policies to ensure regular face-to-face MDT meetings occur when permitted by COVID-19 guidelines.
**Ongoing effort to stimulate true collaboration**	“for me creating a healthier environment is for everybody to actually understand their own worth and understand each other’s worth and that there’s… and that needs to start, probably in like nursing courses and doctor’s courses and then just courses that… because if the hierarchy’s there in the schools and then it’s there in the junior jobs it just sustains it I think” (Med09) “like we did a thing as well before where say we had a group of really difficult patients and there just seemed to be like no result, there seemed to be nothing achievable with them but instead of us all going off to do our own say training needs or you know whatever we needed, we actually did it as a team… it was really, really good because then all of you know rather than the nurses coming saying oh we did a 3 day course in whatever that nobody else knew what we were talking about but we all did it together and kind of brought our brainstorming and that was really good” (Nurse01) “…training development, no matter how small it is, it’s really important for understanding the roles that others have to play… there’s really small you know little tools and strategies you can use- lunches and learns or whatever it is that I think do help” (AHP13)	A more inclusive undergraduate curriculum is needed to weaken traditional status boundaries and improve interprofessional relationships. Ongoing interprofessional training is necessary to promote and maintain collaborative MDT working.

## Discussion

Adopting a narrative inquiry methodology this research provides an in-depth understanding of an under-developed area of study, MDT power dynamics during the COVID-19 pandemic. The findings demonstrate that while a hierarchical team culture prevails in many teams within the Irish health system, perceptions of power and staff realities of autonomy differ across professions. Team characteristics (e.g., staff rotations, MDT meeting structure) were identified as factors mediating the level of interprofessional collaboration possible within MDTs. This research also explored changing perceptions of power during the COVID-19 pandemic. While COVID-19 both supported and hindered interprofessional collaboration, the findings underscore the importance of continued interprofessional education to promote and sustain greater interprofessional care in practice (Table 2).

Many staff portrayed their experiences of MDT power dynamics using military narratives (e.g., first in command, building alliances). Similar combat descriptions have been used in healthcare to describe experiences and attitudes towards disease management and treatment (e.g. battle with cancer, war on HIV). While some authors view the use of military metaphors as empowering [23], others view these narratives as problematic in healthcare contexts [24]. Kraska and Kappeler [25] define militarism as a set of beliefs and values that stress domination as an appropriate response to solve problems and gain power. While most military narratives employed by staff described the negative impact of MDT hierarchies, staffs’ “battle” against COVID-19 appeared to strengthen many MDT relationships particularly in acute care settings. Therefore, task interdependence in fighting a common enemy (i.e. the virus) stimulated greater interprofessional collaboration.

Similar to Price et al. [26] in this research physicians were portrayed as the ‘boss’ and primary decision-makers of the MDT. French and Raven’s [27] Bases for Power Model may explain these findings. This model details several variations of power that enable a leader to exert influence. In this study, staff perceived that the specialist skills, knowledge, and historical dominance of senior physicians [10, 26, 28] enabled these senior team members to exercise expert and legitimate power in their respective MDTs. However, COVID-19 challenged these dynamics. The uncertainty associated with the pandemic response enhanced MDT collaboration as no one individual or discipline was perceived as the expert.

In addition to senior physician expertise, many participants identified the governance of care where patients are admitted under the name of one senior physician as a mechanism reinforcing medical dominance in healthcare. The extant literature confirms that holding legal ownership over patient care preserves physician autonomy, enabling these team members to work independently to decide diagnosis and treatment direction [13, 28]. The findings highlight that during the pandemic this structure of care provision strengthened hierarchical decision-making particularly in community settings. COVID-19 restrictions limited interprofessional communication which excused some senior physicians from gaining contributions from MDT members as these physicians were perceived to have the final say. Physician speciality further influenced how power was exercised. Similar to Nugus et al. [29], the acuity of care (e.g. critical care) and character of the work (e.g. complexity and population) were meditating factors influencing the level of collaboration experienced within MDTs. However, despite the complexity of surgery, these physicians were cited as hierarchical leaders. Literature [30, 31] suggests that their task-oriented focus where influence depends on technical skills rather than interpersonal behaviours may support this decision-making approach. To support the goal of interdisciplinary team-based working in care settings such as the Irish health system [6], the governance of care must be reviewed and policies introduced to encourage interprofessional decision-making irrespective of the local context (e.g. location (i.e. acute vs. community) or physician speciality).

Much of the literature on interprofessional collaboration emphasises the nurse-physician relationship due to the status of medicine and the mass of nurses associated with care provision [26, 32]. As evident in this research, despite the important role of other HCPs in care delivery, many struggle to have their voices heard in terms of patient care decision-making. Similar to Boyce [28], many AHPs perceived their influence as “invisible”. In addition to governance models, most AHPs associated poor interprofessional collaboration with a lack of role clarity. The transient nature of AHP involvement in the MDT (i.e. dependent on patient need) and their physical distance from the MDT (i.e. many located in discipline specific departments and not co-located with the MDT) limits opportunities to meaningfully interact with MDT members [33]. These factors influenced participants’ ability to demonstrate their competence with their fellow MDT members. These findings align with Orchard et al. [34] who emphasises that interprofessional collaboration occurs effectively in contexts with high levels of role understanding and appreciation which builds trust within MDTs. This research exposes how AHPs address these challenges by using collaborative power. Many AHPs negotiated alliances across professions (e.g. nursing and other AHPs) to promote their voice and exercise greater influence within the MDT. However, for community AHPs, pandemic restrictions appeared to further weaken their perceived authority. While some MDT members were categorised as ‘essential’ frontline workers, many community AHPs were mandated to work remotely or redeployed to assist with public health initiatives.

However, perceived and experienced power differed across the MDT. While senior physicians were perceived to hold complete autonomy, for physicians, senior managers assumed dominance in strategic planning [35]. Similar to Russell [36], senior physicians in this research expressed a sense of powerlessness particularly in relation to their inability to implement change without management support. Conversely, for AHPs, while many perceived their influence as limited, acute care staff identified the AHP role as critical in achieving efficient service delivery. For this setting, COVID-19 supported to expose the contribution and value of AHPs within the MDT. As contextual factors such as power structures are dynamic [37], future research should endeavour to explore the evolution of these perceptions among MDTs.

While staff stability and visibility influenced power disparities in all team types, COVID-19 improved interprofessional collaboration among acute care staff through the introduction of ward-based care. Limiting staff rotations and movement through the hospital resulted in heightened familiarity among MDT members which improved role clarity and appreciation across professions. Future research should endeavour to assess the feasibility of implementing ward-based care models to promote greater interprofessional collaboration. While community settings have been previously characterised as more collective [7], by limiting interprofessional communication, COVID-19 restrictions encouraged siloed working, suppressed role understanding and ultimately promoted medical dominance in patient care decision-making.

Most participants emphasised the importance of regular MDT meetings to develop role awareness and trust across professions. Similar to Vogwell and Reeves [38], MDT meetings and huddles positively impacted care coordination and interprofessional communication. However, the findings of this research expose that information exchange rather than nuanced care discussions were the primary focus of most MDT interactions. While this approach improved the efficiency of meetings, it limited staff contribution as their competence and expertise was often overlooked. During COVID-19, strategies to increase social distancing further restricted MDT interactions as communication shifted to virtual platforms (e.g. teleconferencing, videoconferencing). Unlike previous literature [39], the findings reveal that virtual MDT meetings further promoted hierarchical decision-making. A focus on efficiency, the poor engagement of staff, and an inability to read social cues strengthened siloed working in MDTs. To support the implementation of interprofessional care in practice this research recommends the need for regular face-to-face MDT meetings when permitted by COVID-19 guidelines.

The identity of each healthcare profession is reported to be taught and reinforced through the socialisation of staff during their intraprofessional training [40]. Orchard et al. [34] suggests that this socialisation process creates power imbalances on the frontline as intraprofessional learning promotes the development of negative stereotypes. Aligned with previous literature, the findings of this research underscore the importance of interprofessional education to enhance role understanding and appreciation across professions [41]. Therefore, to weaken the traditional status boundaries between professions, we recommend including a more inclusive undergraduate curriculum to improve interprofessional relationships. However, Veerapen and Purkis [42] suggest that high workloads can dissipate the interprofessional collaboration skills acquired during educational programmes. The findings of this research confirm the need for ongoing interprofessional training to promote and maintain collaborative MDT working. While some staff questioned the feasibility of implementing ongoing team training, open-access resources such as the Collective Leadership for Safety Cultures programme [43] can equip staff with the knowledge and skills necessary to communicate and coordinate as a collaborative MDT. However, to support the implementation of interprofessional care in practice, future research is needed to understand where perceptions of power originate in MDTs (i.e., undergraduate training vs. frontline experience). This awareness will support the development of targeted strategies to mitigate power disparities within MDTs.

While this article offers new insights and a greater understanding of MDT power dynamics during the COVID-19 pandemic, some limitations should be noted. Although a diverse sample of HCPs were recruited, the perceptions of some MDT members are missing or underrepresented. Experiences of power among nurses and support staff requires further investigation to assess the relevance of these results across professional groups. The transferability of the findings is further limited as staff self-selected to participate. The sampling approach adopted may have biased the results towards HCPs with extreme experiences of MDT working (i.e. hierarchical vs. collaborative). Additionally, as contextual factors such as power dynamics are influenced by multiple levels of the healthcare system (i.e. system, organisational, team, and individual [37], research across international settings is needed to evaluate the applicability of these findings to wider contexts. However, the contextual descriptions provided by this study enables the reader to interpret the findings and consider their applicability to their team and local setting. To mitigate potential researcher bias, a reflexive journal was maintained, and all researchers were involved in the analysis throughout the evaluation process.

## Conclusion

This study provides a deeper understanding of MDT power dynamics. Despite the impact of power disparities on shared decision-making and collaborative practice, the field’s understanding of this area of study during the COVID-19 pandemic is limited. By exploring staff experiences of power dynamics, this research generated recommendations for future research to support the implementation of greater interprofessional collaboration. By understanding MDT power dynamics, strategies and initiatives can be developed to support HCPs to enter the workforce more prepared for the inevitable teamwork in which they are required to engage, encouraging the implementation of interprofessional care in practice.

## Electronic supplementary material

Below is the link to the electronic supplementary material.

Supplementary Material 1

Supplementary Material 2

## Data Availability

The dataset generated during the current study is not publicly available due to privacy/ethical restrictions. While, the dataset is not available for secondary analysis, a portion of the anonymised/redacted data may be made available on reasonable request from the corresponding author.

## Abbreviations

AHP: Allied health professionals
HCPs: Healthcare professionals
MDT: Multidisciplinary team

## References

[1] Engel J, Prentice D (2013). The ethics of interprofessional collaboration. Nurs Ethics.

[2] Engel J, Prentice D, Taplay K (2017). A power experience: a phenomenological study of interprofessional education. J Prof Nurs.

[3] Apker J, Propp KM, Zabava Ford WS. Negotiating status and identity tensions in Healthcare Team interactions: an exploration of nurse role dialectics. J Appl Communication Res. 2005 May;33(1):93–115.

[4] Tajfrel H (1982). Social psychology of intergroup relations. Ann Rev Psychol.

[5] Ferlie E, Fitzgerald L, Wood M, Hawkins C. The Nonspread of Innovations: the Mediating Role of Professionals. AMJ. 2005 Feb 1;48(1):117–34.

[6] Government of Ireland. Sláintecare Action Plan 2019 [Internet]. Dublin: Houses of Oireachtas; 2019 [cited 2019 Oct 4]. Available from: https://health.gov.ie/wp-content/uploads/2019/03/Sl%C3%A1intecare-Action-Plan-2019.pdf.

[7] Baxter SK, Brumfitt SM (2008). Professional differences in interprofessional working. J Interprof Care.

[8] Hall P (2005). Interprofessional teamwork: Professional cultures as barriers. J Interprof Care.

[9] Weller J, Boyd M, Cumin D. Teams, tribes and patient safety: overcoming barriers to effective teamwork in healthcare. Postgrad Med J. 2014 Mar;89(1061):149–54. 10.1136/postgradmedj-2012-131168 24398594

[10] Braithwaite J, Clay-Williams R, Vecellio E, Marks D, Hooper T, Westbrook M et al. The basis of clinical tribalism, hierarchy and sterotyping: a laboratory-controlled teamwork experiment. BMJ Open 2016;6. 10.1136/bmjopen-2016-012467 PMC4985874 27473955

[11] Braynion P. Power and leadership. J Health Organ Manag. 2004 Jan;1(6):447–63. 10.1108/14777260410570009 15588014

[12] Witz A (1992). Professional and partriarchy.

[13] Baker L, Egan-Lee E, Martimianakis MA, Reeves S (2011). Relationships of power-implications for interprofessional education. J Interprof Care.

[14] Slater B. Lessons from implementation-after the crisis, what then? [Internet]. 2020 [cited 2020 Jul 29]. Available from: https://sites.google.com/nihr.ac.uk/arc-yh/news-events-and-media/blogs/blog-9-beverley-slater.

[15] Bailey S, West M. Learning from staff experiences of Covid-19: let the light come streaming in [Internet]. The King’s Fund. 2020 [cited 2020 Jul 29]. Available from: https://www.kingsfund.org.uk/blog/2020/06/learning-staff-experiences-covid-19.

[16] Wensing M, Sales A, Armstrong R, Wilson P. Implementation science in times of Covid-19. Implement Sci. 2020 Jun;8(1):42. 10.1186/s13012-020-01006-x PMC7276954 32513292

[17] Engum SA, Jeffries PR (2012). Interdisciplinary collisions: bringing healthcare professionals together. Collegian.

[18] Braithwaite J. Between-group behaviour in health care: gaps, edges, boundaries, disconnections, weak ties, spaces and holes. A systematic review. BMC Health Serv Res 2010 Dec 7;10(1):330. 10.1186/1472-6963-10-330 PMC3004899 21134295

[19] Gergerich E, Boland D, Scott MA. Hierarchies in interprofessional training. J Interprofessional Care 2019 Sep 3;33(5):528–35. 10.1080/13561820.2018.1538110 30383437

[20] Wang CC, Geale SK. The power of story: Narrative inquiry as a methodology in nursing research. International Journal of Nursing Sciences. 2015 Jun 1;2(2):195–8.

[21] Braun V, Clarke V (2006). Using thematic analysis in psychology. Qualitative Res Psychol.

[22] Lincoln YS, Guba EG (1985). Naturalistic Inquiry.

[23] Tate TP, Pearlman RA. Military Metaphors in Health Care: Who Are We Actually Trying to Help? Am J Bioeth 2016 Oct 2;16(10):15–7. 10.1080/15265161.2016.1214320 27653391

[24] Cox CL. ‘Healthcare Heroes’: problems with media focus on heroism from healthcare workers during the COVID-19 pandemic. Journal of Medical Ethics. 2020 Aug 1;46(8):510–3. 10.1136/medethics-2020-106398 PMC7316119 32546658

[25] Kraska PB, Kappeler VE (1997). Militarizing american police: the rise and normalization of paramilitary units. Soc Probs.

[26] Price S, Doucet S, McGillis Hall L (2014). The historical social positioning of nursing and medicine: implications for career choice, early socialization and interprofessional collaboration. J Interprof Care.

[27] French J, Raven BH. The bases of social power. Studies in Social Power. Ann Arbor, Michigan:University of Michigan; 1959. 150–67.

[28] Boyce R (2006). Emerging from the shadow of medicine: allied health as a “profession community” subculture. Health and Sociology Review.

[29] Nugus P, Greenfield D, Travaglia J, Westbrook J, Braithwaite J. How and where clinicians exercise power: Interprofessional relations in health care. Social Sci Med 2010 Sep 1;71(5):898–909. 10.1016/j.socscimed.2010.05.029 20609507

[30] Teunissen C, Burrell B, Maskill V. Effective Surgical Teams: An Integrative Literature Review. West J Nurs Res 2020 Jan 1;42(1):61–75. 10.1177/0193945919834896 30854942

[31] Rogers DA, Lingard L, Boehler ML, Espin S, Mellinger JD, Schindler N et al. Surgeons managing conflict in the operating room: defining the educational need and identifying effective behaviors. The American Journal of Surgery. 2013 Feb 1;205(2):125–30. 10.1016/j.amjsurg.2012.05.027 23141805

[32] Reeves S, Nelson S, Zwarenstein M (2008). The doctor–nurse game in the age of interprofessional care: a view from Canada. Nurs Inq.

[33] Seaton J, Jones A, Johnston C, Francis K. Allied health professionals’ perceptions of interprofessional collaboration in primary health care: an integrative review. J Interprofessional Care 2021 Mar 4;35(2):217–28. 10.1080/13561820.2020.1732311 32297811

[34] Orchard CA, Curran V, Kabene S. Creating a culture for interdisciplinary collaborative professional practice. Med Educ Online. 2005;10(1). 10.3402/meo.v10i.4387 28253153

[35] Succi MJ, Lee SYD, Alexander JA (1998). Trust between managers and physicians in community hospitals: the effects of power over hospital decisions. J Healthc Manag.

[36] Russell V, Wyness LA, McAuliffe E, Fellenz M. The social identity of hospital consultants as managers. Journal of Health Organization and Management. 2010 Jan 1;24(3):220–36. 10.1108/14777261011054581 20698400

[37] Rogers L, De Brún A, McAuliffe E. Defining and assessing context in healthcare implementation studies: a systematic review. BMC Health Serv Res. 2020;20(591). 10.1186/s12913-020-05212-7 PMC7322847 32600396

[38] Vogwill V, Reeves S. Challenges of information exchange between nurses and physicians in multidisciplinary team meetings. J Interprofessional Care 2008 Jan 1;22(6):664–7. 10.1080/13561820802114772 19012148

[39] Johnson NA, Cooper RB (2009). Power and concession in computer-mediated negotiations: an examination of First offers. MIS Q.

[40] Hall P, Weaver L (2001). Interdisciplinary education and teamwork: a long and winding road. Med Educ.

[41] McNair RP (2005). The case for educating health care students in professionalism as the core content of interprofessional education. Med Educ.

[42] Veerapen K, Purkis ME. Implications of early workplace experiences on continuing interprofessional education for physicians and nurses. Journal of Interprofessional Care. 2014 May 1;28(3):218–25. 10.3109/13561820.2014.884552 24555541

[43] De Brún A, Anjara S, Cunningham U, Khurshid Z, Macdonald S, O’Donovan R, et al. The collective Leadership for Safety Culture (Co-Lead) Team intervention to promote Teamwork and Patient Safety. Int J Environ Res Public Health. 2020 Nov;17(22):8673. 10.3390/ijerph17228673 PMC7700115 33266448

