# Supplementary material for: Exploring healthcare staff narratives to gain an in-depth understanding of changing multidisciplinary team power dynamics during the COVID-19 pandemic
Source: BMC Health Serv Res. 2023 May 1;23:419. doi: 10.1186/s12913-023-09406-7 (PMC10150666; doi:10.1186/s12913-023-09406-7)
Supplement: Supplementary file 1 — Supplementary Material 1 [file 12913_2023_9406_MOESM1_ESM.docx]

**Interview Topic Guide**

Aim: to gain insight into healthcare teams dynamics and explore whether these dynamics changed in response to the COVID-19 pandemic. Clarify you are interested in exploring experiences working in MDTs and explain what that means

**Background questions**

- Can you tell me a bit about your professional background / time as a X professional?
- Can you tell me a bit about the current team you work in and your current role / responsibilities / duties in this team)?

**Broad- Team dynamics**

In this research team dynamics includes characteristics of the MDT such as communication, trust, respect, influence, and collaboration between MDT members.

- Reflecting on these factors how would you describe the dynamics in your current MDT? What makes you say this (prompt for real world example)
- How do these dynamics differ to other MDTs you have worked in previously? Can you provide examples of how they are different?
- Can you describe an experience that represents a positive example of team dynamics in your current or previous MDT?
  - What was it about how this team worked/operated that made it a positive experience?- who set the tone, how did they do this
- Can you describe an experience that represents a negative example of team dynamics in your current or previous MDT?
  - What was it about how this team worked/operated that made it a negative experience?- who set the tone, how did they do this

**Interpersonal relationships**

- How would you describe the working relationships among members of your current MDT?
  - Do people on the team get on/work well together?
  - Do conflicts occur between team members? Do these conflicts occur between certain disciplines? Would similar conflicts between disciplines occur in other MDTs you have previously worked in?
  - Who are the people you work with most closely in your role? Why is this the case?
  - Has COVID-19 changed the working relationships of MDT members? If so how? Prompt for examples
- What can impact on MDT working relationships? – what would cause working relationships to breakdown?
- What impact do working relationships between MDT members have on:
  - Teamwork
  - Patient care
  - Staff wellbeing

Can you give examples of this impact from your experience?

**Communication**

- How would you describe the communication between members of the MDT? (Prompt: interdisciplinary vs intradisciplinary, vertical vs horizontal leadership)
- Would interdisciplinary communication within this MDT be similar to your past experiences working on other teams?
- Thinking of your current MDT, if an issue or problem arises, how is it typically discussed/addressed/resolved?
- What can impact communication between MDT members?
- What impact does communication between MDT members have on:
  - Teamwork
  - Patient care
  - Staff wellbeing

Can you give me an example of this impact from your experience?

- Has COVID-19 changed communication processes within the MDT? If so how?

**Team roles**

- Do you think the MDT members are clear on their individual roles within the team? Is there clarity on the roles of others on the team?
  - What is the impact of this clarity/lack of clarity on team functioning (conflict between professions)?
- Do you think other MDT members understand (then ask re: appreciate) the role you play in the team?
  - If yes, how do staff show their appreciation- can you provide an example?
  - If no, why do you think this is the case?
- Do you think a similar level of respect is shown to all team members of the MDT regardless of their role?
- Have team roles adapted during the COVID-19 pandemic?
  - Did people adopt different roles/behave differently to how they normally did? Why? Can you give some examples from your experience?

**Psychological Safety/Trust**

- When there is an issue or a problem to address within the MDT, do you feel comfortable to speak up and offer your view? Why do you say that?
  - Do you feel you can voice an unpopular opinion in your team? Do you think all members feel that way?
  - Can you provide an example from your experience?
- Do you feel your view is considered as important as the views of others on the multidisciplinary team?
  - Why do you say that?
  - Can you provide an example from your experience?
  - What impact does this have on: teamwork, patient care, staff wellbeing?
- Would you feel comfortable challenging the opinions of other members of the MDT? Would you feel comfortable challenging some team members more than others?
- When there is a conflict about a decision or how something should be done, who has the final say? Why do you think this the case?

**Influence/Autonomy**

- Who are the strong influencers on the team?
  - Why are these individuals strong influencers?
  - On what kinds of issues do these individuals have influence?
  - Have any new influencers emerged in response to COVID-19 pandemic? If so, why?
  - Do you/others feel listened to and consulted before decisions are made? Why do you say that? Can you provide an example from your experience?
- How would you describe collaboration within the MDT
  - Has this change during the COVID-19 pandemic? Why?

**Future**

- Reflecting on our conversation what do you expect the dynamics of this MDT to look like moving forward following the COVID-19 pandemic?
  - - Do you think any changes in team dynamics will be sustained or will the teamworking return to normal (prior to the pandemic)? Why do you say that?

**Your Say**

Is there anything else that you wanted to mention as important or perhaps expected to be asked about?

(Thank participant and participant debriefing)
